# Quality of life among parents of children with phenylketonuria (PKU)

**DOI:** 10.1186/1477-7525-11-54

**Published:** 2013-03-28

**Authors:** Astrid Fidika, Christel Salewski, Lutz Goldbeck

**Affiliations:** 1 Department of Child and Adolescent Psychiatry/Psychotherapy, University Hospital Ulm, Ulm 89075, Germany; 2 Department of Psychology, University of Hagen, Hagen 58097, Germany

**Keywords:** Parents, Quality of life, PKU, Metabolic disorder, Social support

## Abstract

**Background:**

Parents of children with chronic conditions are known to be at risk of impairment in their quality of life (QoL). Studies considering other chronic conditions proposed diverse factors to have an impact on the parent’s QoL. So far, there has been little research on parents who have a child with phenylketonuria (PKU). This study was designed to evaluate the parental quality of life (PQoL) of parents of children and adolescents who have PKU and identify possible predictors of PQoL.

**Methods:**

In this cross-sectional study 89 parents completed self-report measures of PQoL, family stress, social support, and parental coping. To determine the impact of these potential predictors on PQoL, regression and mediation analyses were performed.

**Results:**

Most parents coped well with their children’s metabolic disorder. Family stress (β = −0.42; p < 0.001) and perceived social support (β = 0.33; p = 0.001) were proven to be the most powerful predictors, accounting together for 45% of the variance of PQoL. Social support mediated the association between family stress and PQoL.

**Conclusions:**

The current study indicates that parents of younger children are an especially vulnerable group. Members of health-care teams should be able to identify and empower vulnerable parents to seek and maintain social support.

## Background

Phenylketonuria (PKU) [OMIM 261600] is an inherited autosomal recessive metabolic disorder caused by a deficiency of the enzyme phenylanalin hydroxylase (PAH, EC 1.14.16.1) with an approximate incidence of 1 per 10,000 newborns in most of the Caucasian populations of northern and western Europe [1]. The nationwide newborn screening in Germany enables early detection of the metabolic dysfunction, so that parents are usually confronted with this diagnosis directly after their child’s birth. Approximately 2,500 children, adolescents and adults with PKU live in Germany. Due to high phenylalanine (phe) concentrations, untreated PKU results in severe irreversible neurological and especially cognitive impairments already within the first year after birth [2]. The treatment consists of a lifelong individually tailored protein-restricted diet, preventing neurotoxic blood phe concentrations [3]. Although PKU can be treated well and normal cognitive development is possible [2], minor neuropsychological deficits may arise [4]. The therapy is very restrictive and demands a high degree of adherence on the part of all family members. It allows only food with a low amount of protein or synthetic products which makes a supplementation of phe-free amino acid mixtures necessary. The amount of consumed protein per day has to be recorded. Therefore parents need to supervise and measure the whole nutritional intake of their child. Altogether, daily dietary management, frequent blood tests for monitoring the phe levels and regular visits to a pediatric centre for metabolic disorder make the therapy complex and require a lot of time and effort from the patients and their families [2]. Especially in the first months and years after diagnosis parents are faced with the fact that PKU presents a serious threat to their child’s development. Reoccurring health-related concerns or treatment adherence problems with the child may constitute chronic stressors for the parents [5]. Lord and colleagues [6] examined parents’ reactions to PKU and showed that a significant proportion of parents (5% of fathers and 12% of mothers) report clinical levels of posttraumatic reactions, even years after diagnosis.

Previous research showed that parents of chronically ill children are at risk of developing psychological disorders and psychosocial problems and to have poorer health than parents of healthy children [7]. However, methodological approaches vary widely in assessing the long term adjustment of parents with chronically ill children. Goldbeck and Storck [8] suggest that Parental Quality of Life (PQoL) may be the most appropriate indicator of parental adjustment. It is defined by physical and psychological well-being and social functioning and covers health-related dimensions like “physical and daily functioning”, as well as dimensions referring specifically to the chronic condition of their child, like “satisfaction with the family”, and “emotional stability”. PQoL differs from the construct of health-related quality of life (HRQoL) in its explicit relation to the chronic condition of the child.

Additionally, PQoL as a psychometric measure enables the identification of parents with sub-clinical impairments due to the chronic condition of their child [9]. In line with this argument, Hatzmann and colleagues [10] compared the PQoL of parents of ill children with parents of healthy schoolchildren and showed that almost half of the parents of chronically ill children are at risk of impairment in their PQoL. Another study also indicated impairment in PQoL among parents with chronically ill children, particularly among parents of children with heart disease [11]. The majority of studies on PQoL involved parents of children with heart disease or other diseases such as atopic dermatitis or pediatric inflammatory bowel disease [12-14]. Considering other multifaceted conditions, such as PKU, much less information about PQoL is available. In a recent Dutch investigation by ten Hoedt and colleagues [15], the HRQoL of parents with children diagnosed with PKU was comparable to that of parents of healthy children and significantly better to that of parents with children diagnosed with other, more life threatening, metabolic disorders.

Although research on PQoL in the context of a child’s chronic condition is increasing, is not fully understood why some parents cope well and others do not. Different factors have been discussed as possible predictors of PQoL. In the investigation mentioned above [15], younger age of the PKU affected child, emotional support and loss of friendship were associated with parental HRQoL or seemed to be predictive of it. Furthermore, studies considering other chronic conditions found that having a younger child may be associated with higher levels of parenting stress and lower PQoL [16]. Coping style has also been identified as an important factor in the adjustment to chronic conditions [17] and turned out to be more predictive of PQoL than characteristics of the child’s handicap [18]. Also, several studies show that a lack of social support negatively affects the adjustment process [19,20], so that social support can be considered as another important influence factor [21]. Accordingly, handling the diet and having consequences for their social life were reported to be the major sources of stress for parents asked about the overall impact of PKU on their lives in a study by Bilginsoy and colleagues [22]. Also, since food is part of many social situations, parents often need support from other family members or their social network to adhere to all of the demands of the diet [6].

The current study has three objectives. First, since there is only little knowledge about adjustment among parents of children with PKU, especially in Germany, the PQoL of this group will be described. The second research objective is to identify predictors of PQoL in the study sample. In several studies PQoL has been linked to family stress, parental coping, and social support. Therefore, these constructs will be included in the analysis, as well as duration of the metabolic disorder and age of parents as potential confounding factors. Since social support has been discussed in other contexts as a potential mediator between parental stress and parental adaptation [23], the third objective is to analyze the role of social support as a possible mediator in the study group.

## Methods

This cross-sectional study was conducted in Germany. Inclusion criteria for parents were: (1) having one or more children with PKU, (2) children and adolescents with PKU being at maximum no more than 20 years old and still living with their parents, (3) PKU having been diagnosed immediately after birth by newborn screening. Parents of children and adolescents who were not diagnosed immediately after birth were excluded from the study. A late diagnosis in conjunction with a late onset of treatment increases the probability of mental retardation, which was anticipated as being a possible confounder with PKU-related stressors for the parents. Participants were recruited during the years 2008 and 2009 at different clinics specialized in therapy for chronic metabolic disorders and from two regional groups of a self-help organization for parents. One clinic is a paediatric rehabilitation centre providing 4- to 6-week inpatient rehabilitation programs for families of children with metabolic diseases. During these programs, the family lives together in an apartment and receives a multimodal program consisting of various treatments (medical, physical and psychosocial). The other two pediatric centres for metabolic disorders provide in- and outpatient medical care for children and adolescents with metabolic disorders.

Eligible parents received a letter explaining the aim of the study, a written consent form, and a questionnaire booklet containing questionnaires about demographic information, family stress due to the metabolic disorder, parental coping, perceived social support and PQoL. The primary caregiver completed the questionnaires. The study was approved by the ethics committee of the University of Applied Sciences Magdeburg-Stendal. A total of 189 parents were asked to participate in the study, of whom 94 (49.7%) completed the questionnaires. Information on reasons for declining participation was not available. After excluding those parents of children who had not been diagnosed directly after birth (*n* = 5), data from 89 parents were available for analysis.

### Measures

Self-report questionnaires were used to collect data on all study variables. Demographic information for the participating parents and their children, collected via a questionnaire developed for the current study, were as follows (Table 1): Age and gender of caregiver and children, marital status of caregiver, employment status of caregiver, education of caregiver, ethnicity, number of children with PKU, and participation in an inpatient family-oriented rehabilitation program.

**Table 1:** Demographic data of the study sample (N = 89)

		**Total sample (*N *=89)**
Gender		
of caregivers	Female	76 (85.6%)
	Male	13 (14.6%)
of children	Female	46 (51.7%)
	Male	43 (48.3%)
Age (years)		
of caregiver	Mean	39
	SD	6.9
	Range	22-59
of children	Mean	9.0
	SD	5.2
	Range	0.8 – 19.2
Ethnicity	Caucasian	88 (98.9%)
caregivers	Other	1 (1.1%)
Marital status	Married	78 (87.6%)
	Divorced/ single parent	11 (12.4%)
Employment status of caregiver	Employed	66 (74.2%)
	Unemployed	23 (25.8%)
Education of caregiver	Lower secondary school/ secondary school (8-10 years)	57 (64.1%)
	General qualification for university entrance/ university diploma (>10 years)	32 (35.9%)

#### PQoL

PQoL was measured with *The Ulm Quality of Life Inventory for Parents of chronically ill children*[8], a 29-item self-report questionnaire specified for parents with a chronically ill child, which is applicable with parents of children with different conditions. It has been developed by factor analysis. This measure consists of five primary scales, which cover five dimensions and a total scale. The dimension physical/daily functioning represents the physical fitness and coping with every day life. The subscale “satisfaction with family” covers the parent’s well-being within the family and relationship and the subscale “emotional stability” covers worries and strain due to the child’s illness. “Self-development” includes aspects of individual free time and chances to express oneself and “well-being” includes sleep, vitality, and depressiveness. Answers are given with regard to the preceding seven days on five-point rating scales ranging from 0 to 4 (‘never’ to ‘always’). In accordance with a convention in the field of PQoL research, all raw scores were linearly transformed to a scale of 0 – 100, where higher scores indicate higher PQoL.

#### Impact on the family

To assess the consequences of PKU for the family, the German version of the *Impact on Family Scale*[24] was used. The American original version by Stein and Riessman [25] differs from this translated version. The German version high scores on subscales indicate that a chronic condition of the child has a large impact on the family. The questionnaire includes 27 items for the impact on the family, and in addition 6 sibling-related items. All items are rated on a four-point scale ranging from 1 to 4 (‘fully correct’ to ‘not correct at al’) by the primary caregiver. General negative impact is covered on five subscales (financial burden, siblings, social relationships, personal stress and problems in coping) and a total score.

#### Parental coping

The German translation of *The Coping Health Inventory for Parents* (CHIP) [26] provided by McCubbin and colleagues [27] was utilized to assess the repertoire of parental coping strategies which are associated with their child’s chronic condition. It is a self-report questionnaire including 45 specific coping behaviours. Parents are asked to indicate how helpful each coping strategy has been in managing the situation.

#### Perceived social support

Social support was assessed by the short form of the German *Social Support Questionnaire- Short Form 22* (FsozU K-22) [28]. This 22-item measure is made up of three subscales, each evaluating a different domain of perceived social support: Emotional support, practical support and social integration. On a five-point rating scale the parents can evaluate whether the items are appropriate to themselves.

### Statistical analyses

All analyses were performed using SPSS version 15.0 for Windows. Missing data were handled using the Expectation-Maximization (EM) estimation method included in SPSS software. This method enables estimation of data by alternating two steps. For each possible completion, probabilities are computed (expectation; E-step) and then the model parameters are reestimated by using the completions (maximazation; M-step). EM is relatively robust and it overcomes some of the limitations of other techniques, such as mean substitution or regression substitution [29].

First, the data were analyzed via descriptive statistics. To prove whether all measures were applicable in the study sample, internal consistency scores (*Cronbach*’s α) were computed for all scales of the questionnaires. Those ranged adequately from 0.67 to 0.92 for the ULQIE scales, 0.77 to 0.93 for the CHIP scales and 0.75 to 0.92 for the scales of the F-SozU. Unfortunately, some of the subscales of the FaBel-questionnaire did show low alphas (α = 0.37 to 0.86). Due to that total scores of the measures were included in further analysis.

No cut-offs for the PQoL-measure were available to distinguish between good and poor PQoL, for that reason quartiles of the PQoL total score were used. The lowest quartile (*Q*_*.25*_), which splits off the lowest 25% of the data, was selected by the authors as the cut-off for being at risk of impaired or poor PQoL. This cut-off was chosen arbitrarily based on existing literature which suggests that about 20 to 30% of parents who have children with chronic conditions require psychosocial support or treatment (e.g., the international TIDES study [30,31].

All participating parents contacted from the different institutions were regarded as one group for statistical analysis because they did not differ in socio-demographic variables or in their PQoL. To examine the effects of socio-demographic data on the dimensions of PQoL, ANOVAs and *Pearson* correlations were computed. For multiple testing, *Bonferroni* was used for post-hoc analyses. Considering the different requirements when caring for a new-born or an almost-adult with PKU, it is problematic to describe the QoL of the parents over this broad range of age. Due to that fact, the following three age groups were compared: Pre-school children (<6 years), school children (6 to 12 years), and adolescents (>12 years).

Multiple regression analysis was performed to identify the impact of the potential predictors on PQoL. The association of some of the socio-demographic data with PQoL was examined in the univariate (pre-) analyses. All possible variables were included in multiple regression analysis. PQoL was included as continuous variable. The decision as to which factors were regarded as most influential in explaining the variance of PQoL was based on beta coefficients and p-values (p < 0.05).

To examine the extent to which social support affects the proposed relationship between perceived stress and the PQoL, a simple mediation model as proposed by Preacher and Hayes [32] was tested. This procedure includes the criteria defined by Baron and Kenny [33] for mediation analysis and is complemented by *Sobel’s* test. First, the predictor should be significantly associated with the proposed mediator, second, the predictor should be significantly associated with the dependent variable, and third, the mediator should be significantly associated with the dependent variable. *Sobel’s* test then addresses whether the total effect of one variable on another is significantly reduced by including a potential mediator [32]. PQoL as dependent or criterion variable, perceived social support as the proposed mediator and perceived family stress as independent variable (predictor) were included to the simple mediation model.

## Results

### Demographic characteristics

Altogether, 13 fathers (14.6%) and 76 mothers (85.6%) participated and nearly all of the parents (*n* = 88, 98.9%) were Caucasian. Parents who had one child with PKU represented 84.3% (*n* = 75) of the sample and 13 parents (14.6%) had more than one child with PKU. A high percentage (*n* = 63; 70.8%) of the families had already participated at least once in an inpatient family-oriented rehabilitation program, the last one being on average 25.3 months ago (*SD* = 41.1 months). Aim of these four-week programs is that the children and their parents are enabled to improve their handling of the PKU-treatment (in terms of medical, dietetic and psychosocial problems) with the help of a multi-professional team. Further demographic data of the caregivers are presented in Table 1.

### Descriptive analysis of parental quality of life in the study sample

Means, standard deviations and range for all domains of PQoL for the total sample as well as quartiles are listed in Table 2. ANOVAs revealed no significant differences between mothers and fathers in their means on all dimensions of PQoL (total score *T* = 0.77, *p* = 0.45; physical and daily functioning *T* = 0.31, *p* = 0.76; Satisfaction with family *T* = 0.27, *p* = 0.79; emotional stability *T* = 0.74, *p* = 0.46; self development *T* = 0.70, *p* = 0.49; well-being *T* = 0.87, *p* = 0.39). Regarding the total score of the ULQIE (*M* = 67.3; *SD* = 14.2) as well as in 4 out of 5 domains, three-quarters of all participating parents reported PQoL scores in the upper part of the scales (0–100). The group of parents classified by the lowest quartile to be at risk for impaired or poor PQoL ranged from 34.4.to 58.5 for the total score (*M* = 47.9; *SD* = 6.6).

**Table 2:** **Means and standard deviations of PQoL scores (ULQIE; *N*** **= 89)**

**Scale**	**Item example (‘In the last 7 days…’)**	**Total sample (*N*** **= 89)**		
		**Mean (SD)**	**Range**	**Quartile**
Physical and daily functioning	‘I felt fit/healthy.’	67.6 (17.2)	3.6-96.4	*Q*_*.25*_ = 57
Satisfaction with family	‘I felt comfortable in my family.’	75.8 (16.9)	20.9-100.0	*Q*_*.25*_ *=* 67
Emotional stabilty	‘I felt burdened due to the illness of my child.’	66.9 (19.8)	12.5-100.0	*Q*_*.25*_ = 50
Self-development	‘I was able to realize wishes and needs.’	52.7 (22.3)	0.0-93.8	*Q*_*.25*_ = 34
Well-being	‘I was active and full of energy.’	73.6 (17.1)	25.0-100.0	*Q*_*.25*_ = 63
Total score		67.3 (14.2)	34.4-92	*Q*_*.25*_ = 59

*Note.* ULQIE-scales were linearly transformed into scales from 0 to 100, where higher scores indicate better QoL, ANOVA revealed no significant differences in means between mothers and fathers (*p* > 0.05). In the table only the *Q*_*.25*_ is listed because the “lowest” quartile was regarded as having impairments in PQoL.

Parents reported that their PQoL was highest in the domain “satisfaction with the family” (*M* = 75.8; *SD* = 16.9) and lowest in “self-development” (*M* = 52.7; *SD* = 22.3). The remaining domains of the PQoL [“physical and daily functioning” (*M* = 67.6; *SD* = 17.2), “well-being” (*M* = 73.6; *SD* = 17.1), and “emotional stability” (*M* = 66.9; *SD* = 19.8)] scored within a small and similar range.

### Association of socio-demographic variables with parental quality of life

Via univariate analyses no significant associations with the dimensions of PQoL could be found for gender and number of the PKU-affected children, parents’ gender, parents’ marital status, employment status, and education. Age of parents was significantly associated with PQoL in the dimensions of “emotional stability” (*r* = .26, *p* = 0.013), “self-development” (*r* = .23, *p* = 0.03) and the total score (*r* = .27, *p* = 0.026). Significant differences between the three age groups on the domain “self-development” (*F* = 13.72, *p* < 0.001) and the “total score” (*F* = 5.71, *p* = 0.005) emerged. Post hoc comparison showed that the “self-development” mean score of parents in the group with children under 6 years (*M* = 39.3, *SD* = 18.7) differed significantly (*p* < 0.001) from parents of both groups with older children (6 to 12: *M* = 63.2, *SD* = 19.2; >12: *M* = 59.7, *SD* = 22.3). Parents of preschool children reported the lowest PQoL total scores (*M* = 61.3, *SD* = 12.9) and differed significantly from the group of parents with school children (*M* = 71.6, *SD* = 15.2; p = 0.011) and from parents of adolescents (*M* = 70.7, *SD* = 12.3).

This indicates that PQoL was the lowest for parents with preschool children, compared to parents with school-aged children and parents of adolescents. Details of the distribution of socio-demographic data by quartiles of the PQoL total score are listed in Table 3.

**Table 3:** **Demographic data by quartiles of PQoL total score (ULQIE; *N*** **= 89)**

**Quartiles of PQoL: ULQIE total score (0-100)**		**<Q**_**.25 **_**(*M *= 47.9; *SD*=6.6; n=22)**	**Q**_**.25**_**- Q**_**.50 **_**(*M *= 63.1; *SD*=2.8; n=22)**	**Q**_**.50**_**- Q**_**.75 **_**(*M *= 72.5; *SD*=2.7; n=21)**	**>Q**_**.75 **_**(*M *= 84.2; *SD*=4.2; n=24)**
Gender					
of caregivers	Male	0	6 (27.3%)	4 (19.1%)	3 (12.5%)
	Female	22 (100%)	16 (72.7%)	17 (80.9%)	21 (87.5%)
of children	Female	13 (59.1%)	15 (68.2%)	7 (33.3%)	12 (50%)
	Male	9 (40.9%)	7 (31.8%)	14 (66.7%)	12 (50%)
Age (years)					
of caregiver	Mean	35.9	38.7	40.9	40.3
	SD	4.7	8.2	7.7	6.3
	Range	27-45	22-54	30-59	29-56
of children	Mean	6.5	8.2	10.5	10.6
	SD	4.6	6.2	5.0	4.0
	Range	1.2-15.5	1.4-19.2	0.8-18.3	3.8-17.7
Ethnicity	Caucasian	22 (100%)	22 (100%)	20 (95.2%)	24 (100%)
caregivers	others	0	0	1 (4.8%)	0
Marital status	Married	20 (90.9%)	17 (77.3%)	19 (90.5%)	22 (91.6%)
	Divorced/single	2 (9.1%)	5 (22.7%)	2 (9.5%)	2 (8.4%)
Employment	employed	14(63.6%)	17 (77.3%)	19 (90.5%)	16 (66.7%)
status of caregiver	unemployed	8 (36.4%)	5 (22.7%)	2 (9.5%)	8 (33.3%)
Education of caregiver	Lower secondary school/ secondary school (8-10 years)	15 (68.2%)	14 (63.6%)	13 (61.9%)	15 (62.5%)
	General qualification for university entrance/ university diploma (>10 years)	7 (31.8%)	8 (36.4%)	8 (28.1%)	9 (27.5%)

### Identification of potential predictors of parental quality of life

Multiple regression analysis was conducted to examine the role of possible predictors such as socio-demographic data (age of children and parents), perceived social support, parental coping, and family stress in explaining the variance of PQoL. For each construct the total score of the measure was included (see Table 4), because low consistencies (*Cronbach’s* α) for some subscales of one measure were found. Overall, the following variables accounted together for about 45%of the variance of PQoL in the study group: age of parents, age of children, social support, family stress, and parental coping. Low family stress (*β* = −0.42; *p* < 0.001), and perceived social support (*β* = 0.33; *p* = 0.001) proved to be the most powerful factors. For the children’s age (*β* = 0.19; *p* = 0.06) trends were found. All other variables did not show a significant association with PQoL.

**Table 4:** Predictors of the study group’s PQoL (final model derived by multiple regression analysis)

**Predictors**	***R***	***R***^***2***^	***F***	**β**	**p**
	0.67	0.45	13.33***		
age of parents				0.04	0.69
age of children				0.19	0.06
social support				0.33	0.001
family stress				-0.42	< 0.001
parental coping				-0.06	0.57

*Note. N* = 89; dependent variable = total score of ULQIE; *** significant at <0.001 level.

### Mediating effect of social support

**Figure 1:**
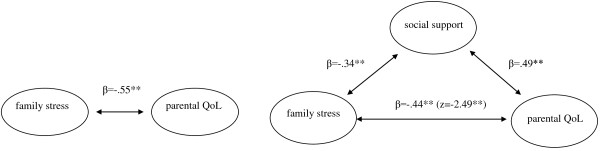
Illustration of the simple mediation model - Perceived social support mediates the association between family stress and PQoL.

Figure 1 illustrates the simple mediation model for testing the proposed mediating effect of social support. The total effect of family stress on PQoL is shown in the first panel of the figure. The second panel describes the effect of family stress on PQoL after controlling for social support. Regression analyses tested first the pathway between family stress and social support (*R*^*2*^ = 0.12, *p* < 0.001, *β* = −.34) and second the pathway between the predictor (family stress) and PQoL as the dependent variable (*R*^*2*^ = 0.30, *p* < 0.001, *β* = −.55). The third step involved testing whether social support as a possible mediating variable was significantly associated with PQoL. The results (*R*^*2*^ = 0.24, *p* < 0.001, *β* = 0.49) indicated a significant positive association between the variable of interest – perceived social support – and PQoL. Hence, the first three conditions were met. *Sobel*’s test achieved significance (*z* = −2.49; *p* < 0.013), indicating that the fourth condition – the significant reduction of the effect of the independent variable (family stress) on the criterion (PQoL) when the mediator (social support) is added – is also fulfilled. This effect is estimated to lie between −0.235 and −0.026 with 95% confidence. Because zero is not included, it indicates that the effect is indeed significant [32].

## Discussion

The current investigation focuses on PQoL among parents of children with PKU. The main aims of the study were to describe PQoL and to consider the extent to which certain factors may be involved in the effect of PKU on PQoL.

### Parental quality of life in the study sample

Considering the stress and the added responsibilities associated with a metabolic disorder, most parents under study were functioning quite well. Due to the low number of participating fathers, a conclusion on differences between mothers and fathers could not be drawn. It could be demonstrated that the majority of parents perceived their quality of life positively, which is similar to the study conducted in the Netherlands showing that the health related quality of life of the parents is almost normal [15]. Within the mentioned study a measure assessing HRQoL was used. In contrast to our study investigating PQoL, which relates to the chronic condition of the child, the used measure relates to the impact of health problems of the parents in general.

Several explanations for the relatively high reported quality of life of the parents are conceivable. Compared to other pediatric chronic or life threatening conditions (diabetes, congenital heart disease etc.), PKU is highly responsive to treatment. Therefore the course and the life expectancy are very promising [2]. Another considerable fact could be that the participating parents are a highly selective group. Most of them participated together with their child in at least one inpatient family-oriented rehabilitation program and received several types of interventions. For parents of children with other chronic conditions (e.g. cystic fibrosis or cardiac disease) these programs are known to improve PQoL [34].

However, in contrast to the general positive response pattern some of the parents reported impairments in their PQoL, especially in their *self-development.* The highest values were perceived on the dimension *satisfaction with the family,* which is consistent with results of other investigations of families with other chronic conditions (e.g. [7,31]). This indicates that they feel comfortable with the support they have from their partner or friends, but feel restricted in developing their personal interests. Regarding differences in PQoL due to the age of the child with PKU it becomes clear that parents of preschool children were mainly affected by impairments of their PQoL in general and specifically in their *self-development*, which is consistent with the results mentioned from the investigations of Streisand and colleagues [16] or ten Hoedt and colleagues [15]. Parents reported to a greater extent higher PQoL when their child attended school and had entered adolescence. Due to the fact that young children need more support and supervision from their parents, especially concerning their nutrition, they are less able to participate actively in the management of their own diet. Getting into school at the age of six or seven, more responsibility for their own diet is required from the children. This and having more routine with the diet years after the diagnosis may lead to more parental freedom and higher PQoL.

### Identification of potential predictors of parental quality of life

In accordance with previous literature suggesting that many different factors mediate the impact of a child’s chronic condition on parental physical and psychosocial health (e.g. [17,21]), we tested the predicting value of variables known to be of importance: Parental coping [17], social support [15,19,20], family stress [35] and demographic characteristics [5] on PQoL. The independent variables in the final regression model explained together about 45%of the variance of PQoL in the study group, with low family stress and perceived social support shown to be most powerful. Furthermore, trends were found for the variable a younger child. These results suggest that the assumptions of the existing literature (e.g. [17,21]) for explaining parental adjustment to a chronic condition of their child are applicable for the group of parents who have a child with PKU.

### Mediating effect of social support

Additionally, results of our study obviously show a mediating function of perceived social support between the perceived stress due to their child’s metabolic disorder and PQoL. This finding implies that the perceived stress resulting from the dietary management is buffered by the social support system. This finding is new for the specific group of parents of children with PKU, but comparable to the results of a study [35] considering another chronic condition. This study investigated the mediating effect of social support between parental stress and parental adjustment, defined as satisfaction with life and psychological well-being, for mothers of school-aged children with cerebral palsy.

### Limitations of the study

Besides the strengths such as the chosen multicentre design there are a few limitations of the current study which should be acknowledged. For example, our sample size was relatively small. Moreover, the fact that no data on reasons for declining participation were available might conceal a self-selection of less or more stressed parents within the study group. This and specific study group characteristics, e.g. that the majority of the participants were married and had already taken part in an inpatient rehabilitation program, leads to the concern that reported PQoL is overestimated. Notwithstanding these limitations, our study group is representative to a certain extent for all parents of children with PKU. Furthermore, the effect that having a younger child is associated with lower PQoL could rather be a more general effect of parenting a child, or a child with a chronic condition, than PKU-specific. The absence of a control group of parents with healthy children or children with other chronic conditions does not allow drawing any conclusion about the specificity for the group of parents of children with PKU. Furthermore, the measure assessing the PQoL has been used for parents of children with PKU for the first time and is validated for other pediatric groups with chronic conditions. Cut-offs for differentiate between poor and good PQoL were not available and are therefore based on the population under study. Additionally, our results solely rely on self-reported measures. Objective data are missing, what contributes to the assumption that the results could be based on other relevant factors we did not assess (e.g. medial characteristics: development of the condition, care-dependency [15,21]). Another limitation concerns methodology: Conclusions regarding causality are not possible based on the cross-sectional design of our study.

## Conclusion and implications for future research and clinical practice

The findings of the present study have several implications for clinical practice. Parents perceive their PQoL positively but may need child-age appropriate support. First of all, the routine assessment of PQoL using standardized questionnaires enables the identification of vulnerable parents and makes it easier for members of the health-care team to offer some extra support for these families, when it is needed. Therefore, the ULQIE with only 29 items and good psychometric properties may be used as a diagnostic screening tool in the care process. The mediating effect of social support is an important finding guiding the design of interventions. Healthcare professionals should not only focus on education about the metabolic disorder, but also should look to empowering parents actively seeking and maintaining social support [21]. Opportunities to strengthen the personal resources of the parents should also be included as relevant factors in such programs [36]. Additionally, it should be mentioned that the current study is one of the first investigations focusing on PQoL related to care-giving for a child with PKU. Further research should include objective data (e.g. development of the condition, care-dependency) and should be based on larger samples as well as longitudinal study designs. It would be interesting to examine whether family-oriented rehabilitation programs could improve PQoL related to PKU and have a protective effect on long-term parental adjustment. To examine the impact of PQoL on treatment adherence would be another interesting issue, because in previous literature different barriers to diet adherence, such as costs of the prescribed food or restrictiveness of the diet, are discussed [2]. Moreover, changes in PQoL during the course of the metabolic disorder and their relation to different stressors in the treatment regime, such as feeding problems, ought to be examined in future prospective studies.

## Competing interests

The authors declare that they have no competing interests.

## Authors’ contributions

AF was the main investigator, collected the data performed data analysis and drafted the manuscript. CS and LG supervised the study and were involved in manuscript drafting. All authors read and approved the final manuscript.
